# Younger age at onset of sporadic Parkinson's disease among subjects occupationally exposed to metals and pesticides

**DOI:** 10.2478/intox-2014-0017

**Published:** 2014-12-30

**Authors:** Marcia H. Ratner, David H. Farb, Josef Ozer, Robert G. Feldman, Raymon Durso

**Affiliations:** 1 Department of Neurology, Boston University School of Medicine, Boston, USA; 2 Department of Pharmacology and Experimental Therapeutics, Boston University School of Medicine, Boston, USA

**Keywords:** occupational exposure, pesticides, metals, Parkinson's disease, onset, age

## Abstract

An earlier age at onset of Parkinson's disease (PD) has been reported to be associated with occupational exposures to manganese and hydrocarbon solvents suggesting that exposure to neurotoxic chemicals may hasten the progression of idiopathic PD. In this study the role of occupational exposure to metals and pesticides in the progression of idiopathic PD was assessed by looking at age at disease onset. The effects of heritable genetic risk factors, which may also influence age at onset, was minimized by including only sporadic cases of PD with no family history of the disease (n=58). Independent samples Student *t*-test revealed that subjects with occupational exposure to metals and/or pesticides (n=36) were significantly (*p=*0.013) younger than unexposed controls (n=22). These subjects were then divided into three groups [high (n=18), low (n=18), and unexposed (n=22)] to ascertain if duration of exposure further influenced age at onset of PD. One-way ANOVA revealed that subjects in the high exposure group were significantly (*p=*0.0121) younger (mean age: 50.33 years) than unexposed subjects (mean age: 60.45 years). Subjects were also stratified by exposure type (metals *vs.* pesticides). These results suggest that chronic exposure to metals and pesticides is associated with a younger age at onset of PD among patients with no family history of the disease and that duration of exposure is a factor in the magnitude of this effect.

## Introduction

Parkinson's disease (PD) is a progressive neurodegenerative movement disorder characterized clinically by tremor, bradykinesia, gait disturbances, cogwheel rigidity, postural instability, hypomimia, hypophonia, and micrographia. The clinical manifestations associated with PD result from the loss of pigmented dopaminergic neurons in the pars compacta of the substantia nigra (Klawans & Cohen, 1970). Although the etiology of PD has not been fully elucidated studies suggest that PD is a multi-factorial disorder, which involves genetic and environmental factors (Tanner & Goldman, 1996; Seidler *et al.*, 1996; Kamel *et al.*, 2014; Tanner *et al.*, 2014; Singh *et al.*, 2014). Although studies have revealed evidence for familial forms of PD a genetic factor has not been identified in the majority of cases. As a result, the possible role of environmental and occupational exposures to neurotoxicants in the development of idiopathic PD has received considerable attention from the medical and public health communities (Ballard *et al.*, 1985; Pezzoli *et al.*, 1995; Tanner & Goldman, 1996; Seidler *et al.*, 1996; Menegon *et al.*, 1998; Savolainen *et al.*, 1998; Smargiassi *et al.*, 1998; Saunders-Pullman *et al.* 1999; Feldman & Ratner, 1999; Priyadarshi *et al.*, 2000; Petrovitch *et al.*, 2002; Dawson & Dawson, 2003).

Environmental factors that have been associated with an increased risk of developing PD include pesticide exposure, rural living, well water consumption, and diet (Rajput *et al.*, 1987; Barbeau, 1987; Calne *et al.* 1987; Liou *et al.*, 1997; Smargiassi *et al.*, 1998; Kuopio *et al.*, 1999; Petrovitch *et al.*, 2002). Despite decades of research, exposure to a specific neurotoxic chemical has never been shown to increase the incidence or prevalence of idiopathic PD. Research has however begun to accumulate, suggesting that exposure to neurotoxic chemicals can influence age at onset of PD. Racette *et al.* (2001) found a significantly younger age at onset of PD among manganese-exposed welders. The mean age at onset among these welders was 46 years while the unexposed controls had a mean age at onset of 63 years (*p<*0.0001). A similar study by Pezzoli *et al.* (2000) which looked at hydrocarbon exposure also revealed a younger age at onset among exposed subjects with PD. The neurotoxic substances most frequently encountered by the exposed subjects were acetone, 2-di-methyl-ethyl-ketone, n-hexane and its isomers, cyclo-hexane and its isomers, hepthane and its isomers, ethyl-acetate, isobutylacetate, butyl-acetate, dichloropropane, trichloroethylene, trichloroethane, tetrachloroethylene, freon, toluene, and 1-methoxy-2-propanol. The exposed group had a mean age at onset of 55.2 years (±9.8 years) compared with 58.6 years (±10 years) for unexposed controls. The severity of symptoms was correlated with the duration and the intensity of exposure to hydrocarbons. In addition, the exposed subjects were less responsive to treatment and required a higher mean dosage of levodopa than did the unexposed controls. These findings were interpreted to suggest that hydrocarbon exposure might be involved in the pathogenesis of PD, which does not appear to have a major genetic component. These observations are important because these data provide evidence for a scientifically plausible interaction between a genetic predisposition and environmental risk factors. However, although Pezzoli *et al.* (2000) and Racette *et al.* (2001) have both provided evidence that age at onset of PD is influenced by occupational exposure to chemicals neither of these studies took specific measures to minimize the opportunity for interactions between exposure and known heritable genetic risk factors which may also influence age at onset and thus, it is difficult to interpret these findings in relation to sporadic PD. Although Racette *et al.* (2001) reported that family history of PD was similar among exposed subjects and unexposed controls, over 50% (n=8) of the 15 exposed subjects included this study had a positive family history of PD making the influence of genetics on age at onset in this relatively small sample population particularly difficult to interpret. Pezzoli *et al.* (2000) also reported that family history of PD was similar (approximately 25%) among exposed subjects and controls but again the influence of genetics on these data is difficult to interpret since subjects with a positive family history for PD were not excluded from the study or by stratification during data analysis.

To minimize the influence of genetic factors on age at onset of PD while further elucidating on the role of chemical exposure history we elected to look at age at onset among subjects with no family history of the disease (sporadic PD). It is hoped that this approach will not only reveal effects of exposure that could be masked by dominant genotypes but may also provide valuable insight that will be useful in the development of novel therapeutics that may be more beneficial for treating sporadic PD.

## Methods

### Human subjects

Human subjects and controls (n=58) participating in this study were drawn from the clinical population of patients with PD seen at the Movement Disorder Center at the Boston University Medical Center, Boston, Massachusetts. All subjects signed a Human Subjects Committee consent form. All subjects included in this study reported having no family history of PD among their first-degree relatives. These subjects were initially deemed eligible for the GenePD study (Maher *et al.*, 2002) but were later excluded because of a negative family history of PD among their first-degree relatives. No effort was made to include or exclude subjects based on gender or race. The diagnosis of PD was confirmed by a clinical evaluation by a Board Certified Neurologist specializing in movement disorders. Every subject included in this study met the Ward and Gibb diagnostic criteria for selection of subjects for research in PD (Ward & Gibb, 1990; Taylor *et al.*, 1999; Maher *et al.*, 2002) (Table 1).

**Table 1:** Occupations associated with exposures to metal and pesticides. Subjects were asked if they had ever worked in any of the following industries with recognized risk for occupational exposure to metals of pesticides.

Metals	Pesticides
Chemical manufacturing	Farm ranch or orchard
Metal finishing industry	Landscaping
Lumber or wood manufacturing	Forestry, paper mill, or woodworking
Electroplating	Rodent control
Battery manufacturing	Weed control
Glass, stone, or clay manufacturingr	Crop dusting
Paper or pulp manufacturing	Pesticide manufacturing
Foundry	Pesticide applicator
Autobody repair	
Smelter	

A trained research assistant interviewed each subject about their occupational exposure to chemicals and recorded the information obtained during the interview on a structured questionnaire form as previously described (Maher *et al.*, 2002). The subject was questioned by the research assistant about whether he or she had worked for more than six months (cumulative exposure) in various industries with a recognized risk for occupational exposure to metals or pesticides. If the subject affirmed having worked in a specific industry, then he or she was asked about his or her history of exposure to specific neurotoxic compounds commonly encountered by workers employed in these industrial settings (Tables 1 and 2).

**Table 2:** Potential occupational chemical exposures in various industries. List of metals and pesticides exposures inquired about and recorded on questionnaire by interviewer. Specific examples of common insecticides, herbicides, fungicides, and rodenticides are provided.

Metals	Pesticides
Mercury	Insecticides
Copper	*Organochlorines (DDT)*
Zinc	*Carbamates (Sevin)*
Manganese	*OPCs (chlorpyrifos)*
Iron	*Pyrethroids*
Magnesium	Herbicides
Lead	*Paraquat*
Other metals	Fungicides
	*Maneb*
	Rodenticides
	*Diphacinone*

### Categorization of exposed cases and unexposed controls

#### Exposed subjects

Subjects with no family history of PD who reported to have experienced at least six months of occupational exposure to metals and/or pesticides were included as exposed subjects in this study (n=36). Subjects reported their occupational exposure histories to a trained interviewer who recorded specifics about the nature of the exposure circumstances in a narrative note and categorized the subject's exposure circumstances based on the following four objective grouping criteria obtained from the questionnaire form used for the GenePD study (Maher *et al.*, 2002).

1. Less than once a month for less than 10 years.
2. Less than once a month for 10 years or more.
3. Once a month or more for less than 10 years.
4. Once a month or more for 10 years or more.

A maximum number of possible exposure days were calculated for each exposure category using these definitions (Table 3).

**Table 3:** Mathematical calculations showing differences in maximum possible days of occupational exposure for subjects in each category.

Exposure category	Maximum possible exposure *
Category 1	11 days × 9 years = max. exposure = 99 days
Category 2	11 days × 10 years = max. exposure >110 days
Category 3	13 days × 9 years = max. exposure >117 days
Category 4	13 days × 10 years = max. exposure >130 days

* Assumes that subjects exposed less than once per month were not exposed more than 11 times per year and that subjects exposed more than once per month were exposed at least 13 times per year by definition. Likewise, assumes that subjects exposed for less than 10 years were not exposed for more than 9 years and subjects exposed for 10 years or more exposed for a minimum of 10 years by definition.

To determine if exposure duration influenced age at onset of PD we constructed a two–tiered exposure duration index based on the exposure data obtained by the interviewer. For the purposes of this study a low-level exposure was defined as that which occurred less than once per month for at least six months but for less than 10 years (category 1 from questionnaire). For example, one of the subjects in this category reported “light exposure” to insecticides, herbicides, fungicides, and rodenticides while working with stored grain and agricultural products at age 10. This low level exposure category was deemed the most likely to select for those subject's who had worked briefly either as teenagers or during career changes in occupational settings where there was a risk for occasional exposure to metals and pesticide. Exposure categories 2 though 4 from the questionnaire were defined as high-level exposures. For example, one subject in this category reported working as an autobody repair mechanic for 3 years with exposures to lead more than once per month. This grouping pattern was deemed the best for identifying those subjects who had either worked in occupations where overt recognized exposures occurred relatively frequently (more than once per month) or had worked for more than 10 years in careers that involved the risk for recognized occasional exposures and the potential for unrecognized exposures. The interviewer's narrative notes were used to aid in categorizing the subject's exposure level and in determining the average duration of exposure for subjects in these two groups.

#### Unexposed controls

Subjects with no family history of PD and no history of occupational exposure to heavy metals and/or pesticides were assigned to the control group (n=22).

### Data analysis

Data was analyzed using SPSS software installed on a Macintosh computer (Apple Computer, Cupertino, CA) at 95% confidence intervals (*p=*0.05). Independent samples Student *t*-test was used for analysis of the effects of gender and smoking history on age at onset of PD. One-way analysis of variance (ANOVA) was used to ascertain if duration of exposure significantly influenced mean age at onset among the three groups of PD subjects stratified by levels of exposure to neurotoxic chemicals. ANOVA was also used to determine if age at onset of PD among subjects and controls was influence by exposure to metals or pesticides. Post Hoc analysis of ANOVA data were performed using Tukey's HSD and the Least Significant Difference (LSD) tests The Pearson correlation coefficient was used to determine if age at onset of PD was correlated with duration of occupational exposure.

## Results

The mean age at onset of PD among the entire sample population (n=58) studied was 55.4 (range 34 to 80 years; SD±10.95). Analysis of skewness and kurtosis indicated that the sample population had a normal distribution (data not shown).

**Figure 1:**
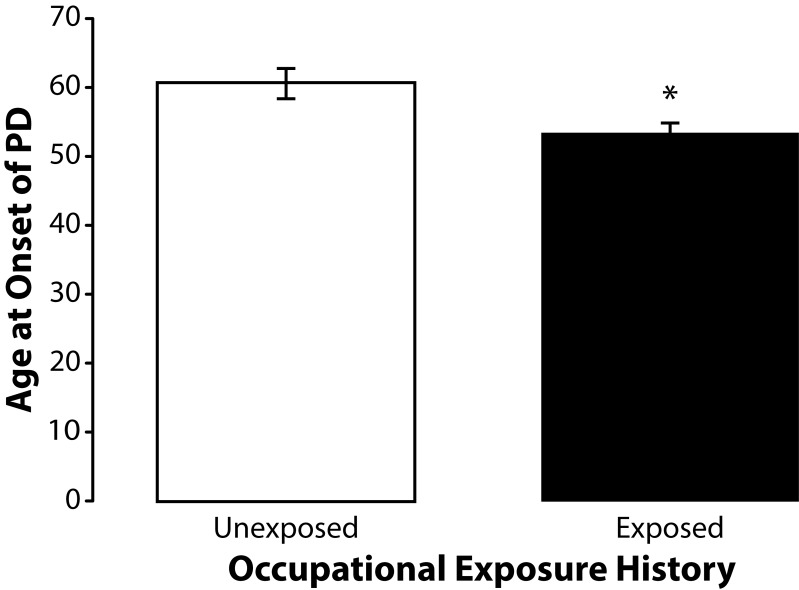
Means and standard errors of the means for age at onset of PD among occupationally exposed (n=36) and unexposed (n=22) subjects. The exposed subjects were significantly younger (t=2.5634; df=56; *p=*0.013) than the unexposed controls.

The *a priori* hypothesis for this study was that occupational exposure to neurotoxic chemicals such as heavy metals and pesticides influenced age at onset of PD among subjects with no family history of the disease. To ascertain if occupational exposure history influenced age at onset of PD we compared exposed subjects with unexposed controls. The exposed subjects (n=36) were younger with a mean age at onset of PD of 53.09 years (SD±10.29). The unexposed controls (n=22) had a mean age at onset of PD of 60.45 years (SD±10.71). Importantly, independent samples *t*-test revealed that the difference in age at onset of PD in these two groups was significantly different (t=2.5634; df=56; *p=*0.013) (Figure 1).

**Figure 2:**
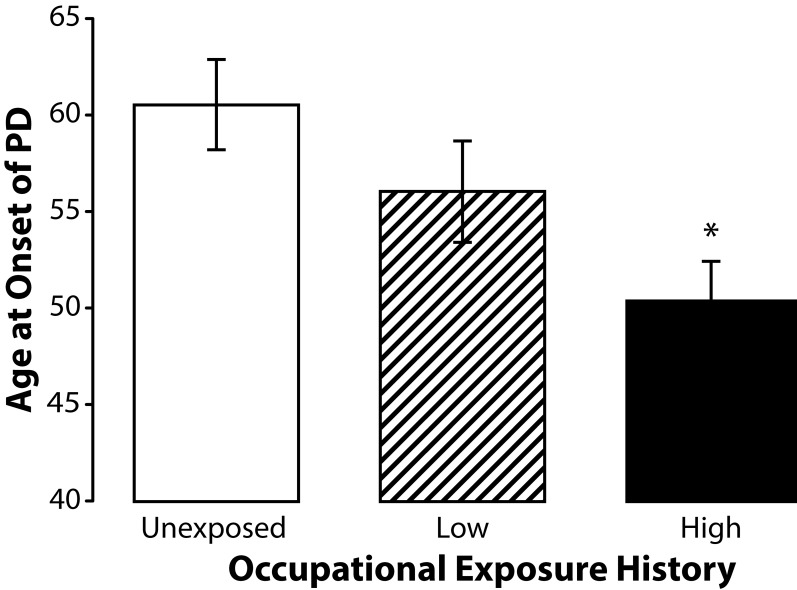
Means and standard errors of the means for age at onset of PD among subjects in high (n=18) and low (n=18) exposure groups and unexposed controls (22). Subjects in the high exposure group were significantly younger (F=4.7913; *p=*0.0121) than the unexposed controls.

To determine if age at onset of PD was further influenced by duration of exposure we stratified our subjects into three groups based on self-reported occupational exposure history. A two-tiered exposure duration index was used to stratify exposed subjects into high and low exposure groups. A low-level exposure was defined as that which occurred less than once per month for at least six months but for less than ten years. A high-level exposure was defined as that which occurred either: A) less than once per month for ten years or more; B) more than once per month for at least six months but for less than ten years; or C) more than once per month for ten years or more. The mean age at onset among subjects in the high-level exposure group which consisted of those subjects who were more likely to have held full-time professional positions working with metals or pesticides for several years or more was 50.33 years (SD±8.75). The mean age at onset among subjects in the low exposure group, which consisted primarily of subjects who briefly worked with metals or pesticides while young adults, was 56 years (SD±11.15). The mean age at onset among unexposed controls was 60.45 years (SD±10.71). The range for ages at onset among the subjects in the high-level exposure group was 34 to 66 years with a median of 50.33 years. This is in contrast to the range of 45 to 80 years with mean age at onset of 60.45 years among the unexposed controls. ANOVA of the age at onset of PD data from subjects stratified into three groups (high, low and unexposed) at 95% confidence intervals using Tukey's HSD for post hoc comparisons revealed a significantly (F=4.7913; *p=*0.0121) younger age at onset of PD among subjects from the high exposure group suggesting that duration and frequency of exposure influences age at onset of PD (Figure 2). Independent samples *t*-test indicated that age at onset of PD among subjects in the low exposure group did not significantly differ (*p=*0.207) from that of the unexposed subjects in this study.

**Figure 3:**
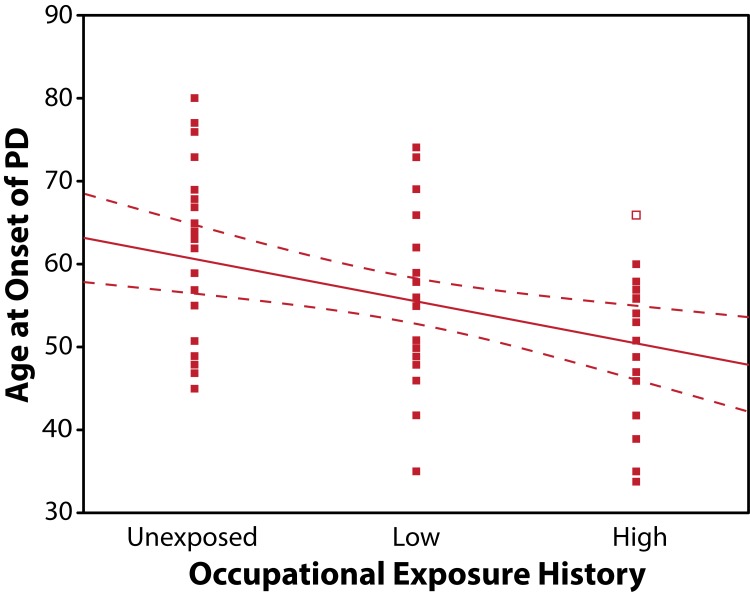
Scatter plot and regression line with confidence bands demonstrating the negative correlation between exposure history and age at onset of PD (r=–0.384; *p=*0.003).

There was a significant (r=–0.384; *p=*0.008) negative correlation between age at onset of PD and duration of occupational exposure (Figure 3). By squaring of the absolute value of the correlation coefficient we derived the coefficient of determination (R^2=^0.147) for these data which indicates that only about 15% of the variance in age at onset of PD seen in this sample population can be explained by duration of exposure. This finding indicates that other factors such as magnitude or intensity of exposure to metals and pesticides may have also influenced the younger age at onset seen in this population.

**Figure 4:**
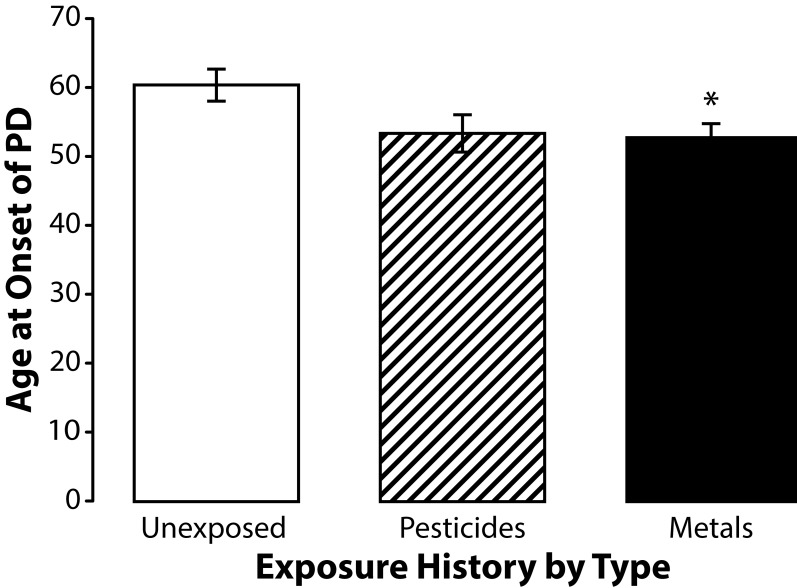
Means and standard errors of the means for age at onset of PD among subjects exposed to metals (n=19), subjects exposed to pesticides (n=17), and unexposed controls (n=22). Post hoc analysis using the Least Significant Different test revealed that subjects exposed to metals were significantly younger (F=3.2848; df=56; *p=*0.045) than unexposed controls. Subjects exposed to pesticides were younger than the unexposed controls but this strong trend difference did not reach statistical significance (*p=*0.057).

**Figure 5:**
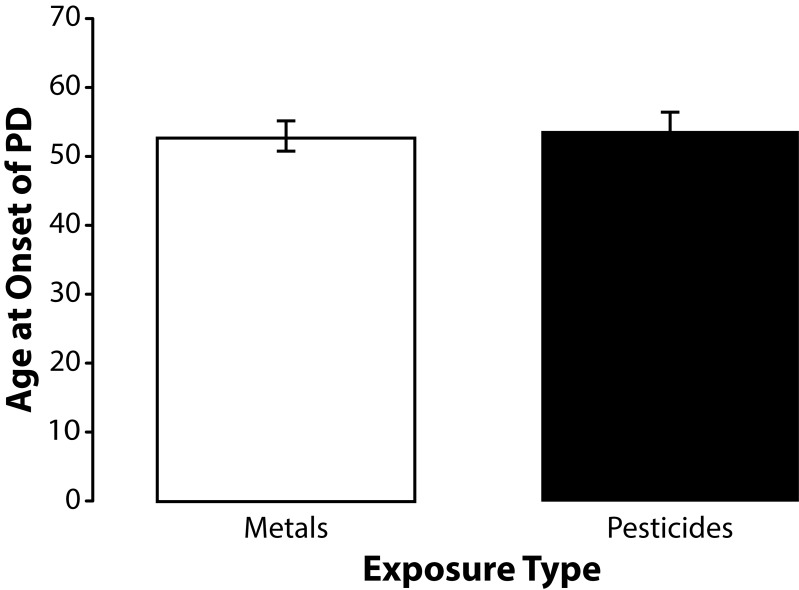
Means and standard errors of the means for age at onset of PD among subjects exposed to metals (n=19) and pesticides (n=17). The difference in mean age at onset of PD among the subjects in these two groups did not even approach statistical significance (t=0.1974; df=34; *p=*0.845).

To better understand the influence of pesticide and metal exposures on age at onset of PD we stratified subjects by their pesticide and metal exposure histories. Although few subjects reported exposure to only one chemical, were able to stratify subjects by their primary exposure. Forty-seven percent (17/36) of the occupationally exposed subjects participating in this study reported occupational exposure to pesticides the remaining were exposed to metals. ANOVA using the Least Significant Difference (LSD) test for post hoc analysis revealed that subjects who reported occupational exposure to metals had a significantly (*p=*0.045) younger age at onset of PD (mean age at onset 52.84 years; SD±9.66 years) than the unexposed controls (mean age at onset 60.45 years; SD±10.71 years) (Figure 4). Subjects exposed to primarily to pesticides were younger than unexposed controls with a mean age at onset of 53.53 years (SD 11.23 years) but his difference was not statistically significant (*p=*0.057). The mean age at onset of PD among subjects exposed to metals and pesticides was very similar and did not approach statistical significance (Figure 5).

**Figure 6:**
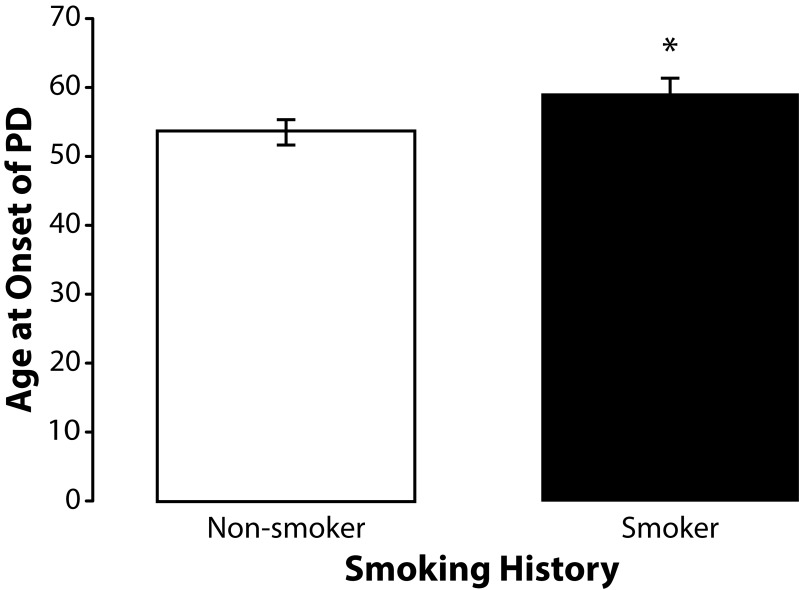
Effects of smoking on age at onset of PD. Means and standard errors of the means for age at onset of PD among former non-smokers and smokers. Former smokers (n=26) are significantly (t=2.0250; df=56; *p=*0.048) older than non-smokers (n=32).

**Figure 7:**
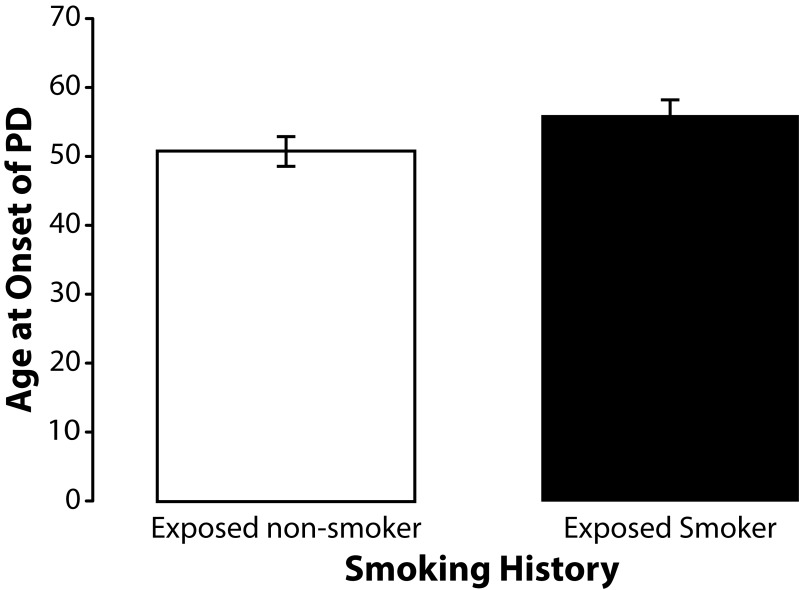
Means and standard errors of the means for age at onset of PD among former occupationally exposed non-smokers and smokers. Although former exposed smokers (n=17) were older at onset of PD than the nonsmokers (n=19), the difference in age at onset of PD was not statistically significant (t=1.4914; df=34; *p=*0.145).

**Figure 8:**
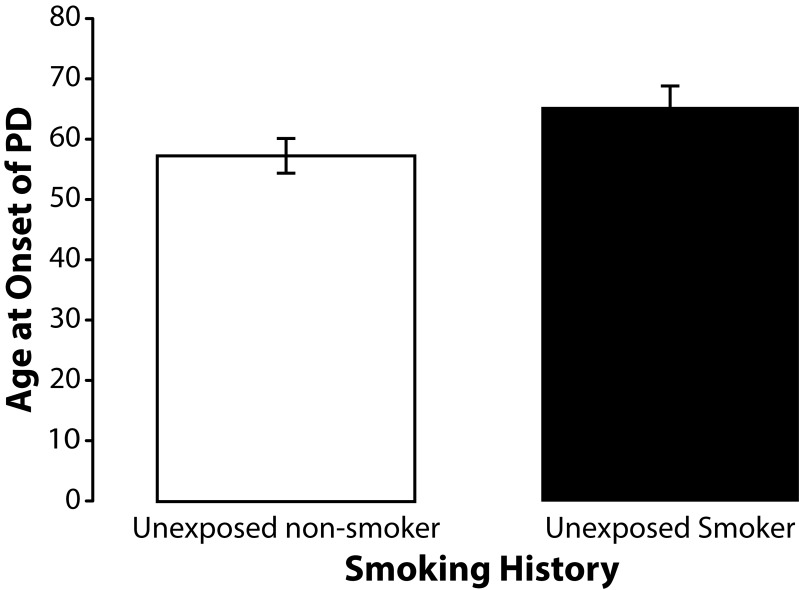
Means and standard errors of the means for age at onset of PD among unexposed non-smokers and smokers. Although the unexposed smokers (n=9) were slightly older at onset of PD than the unexposed nonsmokers (n=13), the difference in age at onset was not statistically significant (t=1.8329; df=20; *p=*0.082).

To determine if smoking history, which has previously been associated with a decrease in the incidence of PD (Smargiassi *et al.*, 1998; Taylor *et al.*, 1999), had any influence on age at onset of sporadic PD among the subjects participating in this study we stratified subjects by smoking history. None of the subjects in this study currently smoked. Smoking history was similar for unexposed (40%) and exposed subjects (47%). The mean age at onset of PD among former unexposed smokers (n=26) was 59.08 (SD±11.31). The mean age at onset of PD among former exposed smokers (n=32) was 53.38 (SD±10.12). Independent samples *t*-test revealed that this difference was significant (*p=*0.048) (Figure 6). Occupationally exposed former smokers (n=17) were also older with mean age at onset of 55.82 years (SD±10.75) than the exposed non-smokers (n=19) who had a mean age at onset of 50.79 years (SD±9.50) but this difference was not statistically significant (*p=*0.145) (Figure 7). A similar effect was found among unexposed nonsmokers (n=13) who were also younger than unexposed former smokers (n=9) but again this difference did reach statistical significance (*p=*0.082) (Figure 8). We also found that the percentage of former smokers in the low exposure group was 61% (11/18) which was nearly double the percentage of former smokers in the high exposure group 33% (6/18) suggesting that some of the variance in age at onset of PD among subjects in the high and low exposure groups may have been due to smoking history.

**Figure 9:**
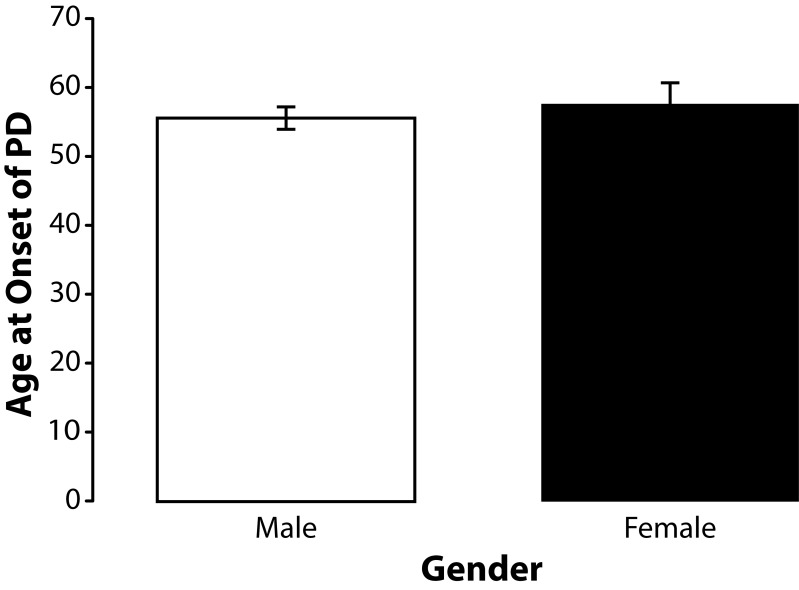
Effects of gender on age at onset of PD. Means and standard errors of means for age at onset of PD male (n=45) and female (n=13) subjects with PD. Although age at onset for the males was slightly younger than that of the females in this study the difference was not significant (t=0.5687; df=56; *p=*0.572).

Because the majority of subjects in this study were men we elected to determined if gender had any influence on age at onset PD. The sample population (n=58) included 13 women and 45 men. The mean age at onset of PD among the male subjects (n=45) was 55.49 years (SD±11.03 years). The mean age at onset of PD among the female subjects (n=13) was 57.46 years (SD±10.98 years). Independent samples *t*-test indicated that gender did not significantly (*p=*0.572) influence age at onset among the subjects in this sample population (Figure 9).

Eleven of the subjects included in this study reported experiencing a loss of consciousness prior to the onset of their symptoms of PD. The age at onset among those subjects with a positive history for loss of consciousness was 59 years (SD±13.53). By contrast, the mean age at onset among the 45 subjects with a negative history for loss of consciousness was 55.2 years (SD±10.30). These findings indicate that loss of consciousness did not account for the younger age at onset found among the subjects with sporadic PD included in this study.

## Discussion

### Exposure history

The results of this study suggest that occupational exposure to metals and/or pesticides is associated with a significantly younger age at onset of idiopathic PD among subjects with no family history of the disease among their first-degree relatives. This observation is particularly important because in contrast to previous studies the data presented here indicate that the effect of occupational exposure to chemicals on age at onset of PD is significant even when the influence of heritable genetic factors is minimized by excluding subjects with a positive family history of the disease (Pezzoli *et al.*, 2000; Racette *et al.*, 2001).

To ascertain the effect of duration of exposure on age at onset of sporadic PD we stratified our subjects by duration of occupational exposure. Subjects in the high exposure group, which consisted of those subjects with longest duration of occupational exposure, were found to be significantly younger than the unexposed controls (*p=*0.0121). Although the subjects in the low exposure group were slightly younger than the unexposed controls, this difference was not statistically significant (*p=*0.207). Duration of occupational exposure to metals and/or pesticides negatively correlated with age at onset of PD in this study. However, the coefficient of determination indicated that only 15% of the variance in age at onset of PD was associated with exposure duration in this study. This observation indicates that other factors such as intensity or type of exposure and genetic factors that influence detoxification of neurotoxicants may also play an important role in age at onset in exposed subjects with sporadic PD. It is important to note that herbicide exposure has been shown to modify the relationship between glutathione-*S*-transferase pi gene polymorphisms and age at onset in familial PD (Wilk *et al.*, 2006).

Estimating the level of exposure over a lifetime is difficult using historical exposure data obtained via questionnaire because this method does not provide for access to the actual occupational exposure levels necessary to ascertain the influence of exposure intensity on the outcome measured (Fidler *et al.*, 1987). The use of duration of exposure, which inherently ignores intensity of exposure, has its limitations but this parameter has nevertheless been used in many other studies (Hane *et al.*, 1977; Harkonen *et al.*, 1978; Lindstrom & Martelin, 1980; Seppalainen *et al.*, 1980; Iregren, 1982; Olson, 1982). These limitations of duration as the sole parameter are most likely reflected in our data, which suggest that 85% of the variance in age at onset PD seen in this study can be attributed to variables other than duration of exposure. These findings indicate the need for additional research using subjects with well-documented exposure history that will permit stratification by intensity as well as duration of exposure.

Analysis of the influence of exposure type on age at onset of PD in this study was conducted by stratifying our subjects into two groups based on whether they had been exposed primarily to metals or pesticides. Subjects who reported occupational exposure to metals were significantly younger than unexposed controls (*p=*0.045). Subjects exposed to pesticides were also younger than controls and that although this difference did not reach statistical significance (*p=*0.057) – there was a very strong trend. Comparison of mean age at onset of PD among subjects exposed to metals with that of subjects exposed to pesticides also failed to reveal a significant difference (*p=*0.845).

Previous reports suggest that subjects exposed to manganese (mean: 46 years) have a younger mean age at onset than subjects exposed to hydrocarbons (mean: 55.2 years). However, comparing the data sets from these two studies in an attempt to arrive at any conclusions about the effects of a specific chemical on age at onset of PD is difficult since the exposure levels as well the specific type of chemical exposure may have varied considerably (Pezzoli *et al.*, 2000; Racette *et al.*, 2001). Although our findings also suggest that metal exposure may have a greater influence on age at onset than pesticide exposure additional research is nevertheless needed to ascertain how exposure to specific chemicals may differentially influence age at onset of sporadic PD.

### Other factors possibly influencing age at onset of PD

#### Gender

Although men out numbered women in this study, gender did not significantly influence age at onset of sporadic PD among subjects occupationally exposed to metals and/or pesticides. These data in subjects with sporadic PD are also consistent with the findings of Maher *et al.* (2002) who reported no difference in mean age at onset of PD among male and female siblings with PD. However, it should be noted that Maher *et al.* (2002) did find that females were more likely to have a first-degree relative with PD, suggesting that male subjects are more likely to be afflicted with sporadic PD. Since our subjects were derived from the same population this pattern may account for the ratio of males to females in our entire sample population of subjects with sporadic PD. The ratio of exposed males to females seen in this study is also consistent with other studies, which too have reported that men are more likely to have a history of occupational exposure to hydrocarbons and manganese than women (Pezzoli *et al.*, 2000; Racette *et al.*, 2001). More work is needed to determine how gender interacts with exposure history and genetics to influence age at onset of PD among subjects with sporadic and familial PD.

#### Smoking behavior

The results of this study revealed a significantly (*p=*0.048) older age at onset of PD among former smokers suggesting that smoking may provide a protective effect against the preclinical progression of sporadic PD. The effect of smoking on age at onset PD was opposite that of occupational exposure to metals and pesticides. Although smoking history was associated with an older age at onset, it most likely did not account for the older age at onset seen among the unexposed subjects in this study because smoking history was similar for the unexposed and exposed subjects. In fact, exposed subjects were actually more likely to have smoked than were unexposed subjects (47% versus 40% respectively) and therefore more likely to have been afforded the protective benefits of smoking.

The reduced risk for PD among smokers is fairly well documented but the effect of smoking on age at onset of sporadic PD is less clear (Smargiassi *et al.*, 1998; Taylor *et al.*, 1999; Hernan *et al.*, 2001; Petrovich *et al.*, 2002; Maher *et al.*, 2002). No association between age at onset and smoking history (smoked versus never smoked) has been observed in the GenePD study suggesting that the protective effect of smoking may greater in sporadic PD than in familial PD (personal communication, Jemma Wilk).

Although inhibition of monoamine oxidase B (MAO B) is suspected to be involved in the reduced risk of PD, the mechanism by which smoking could slow the progression of PD has not been fully elucidated. MAO B polymorphisms such as the G to A transition in intron 13 have been shown to interact with catechol-O-methyltransferase (COMT) polymorphisms to influence relative risk for PD (Wu *et al.*, 2001). However, the MAO B intron 13 polymorphism does not appear to have a major role in the risk for PD, either by itself or by interacting with smoking (Hernan *et al.*, 2002). Additional research is needed to further elucidate on the role of these observations in age at onset of sporadic PD.

#### Prior head trauma and loss of consciousness

History of loss of consciousness prior to onset of PD was reported among eleven of the subjects included in this study. The age at onset among subjects in this study with a positive history for loss of consciousness was 59 years. By contrast, the mean age at onset among the 45 subjects in this study who reported a negative history for loss of consciousness was 55.2 years indicating that loss of consciousness could not possibly have accounted for the younger age at onset of PD we observed among the occupationally exposed subjects in this study.

The relationship between prior head trauma and/or loss of consciousness and risk for developing PD is unclear. A study by Factor and Weinstein (1991) evaluated the relationship between head trauma, loss of consciousness, and PD among 97 patients with PD. Sixty-four spouses served as controls. Thirty-one PD patients reported head trauma before onset of PD whereas only 11 controls reported head injury before completing the study survey. Twenty PD patients and five controls reported head injury associated with alteration or loss of consciousness. Injury occurred a mean of 37.7 years before onset of PD and 37.2 years before survey completion in the two groups, respectively. No significant differences were found between the two groups after controlling for sex. However, a trend toward significance was observed when examining head trauma with alteration of consciousness.

Other studies have found an association between a younger age at onset of PD among subjects who reported a history of head trauma and/or loss of consciousness prior to disease onset (Taylor *et al.*, 1999). Maher *et al.* (2002) found an association between head trauma and a younger age at onset of PD among a sample of 203 siblings pairs diagnosed with PD suggesting that factors such as head trauma which may contribute to a global loss of neurons throughout the central nervous system (CNS) as well as neuronal loss in the basal ganglia and substantia nigra may influence age at onset of PD.

Studies looking at young onset (<40 years old) PD patients found that head injury and exercise were the significant predictors of risk. Keeping all other variables constant, head injury was a risk factor and exercise appeared to be a protective factor. These findings suggest that head trauma may trigger and expedite the appearance of parkinsonian features, but such acceleration may be prevented through regular exercise (Tsai *et al.*, 2002). More research is needed to determine how occupational exposures to neurotoxic chemicals and prior head trauma or loss of consciousness may interact to influence age at onset of sporadic PD.

#### Genetics

This research has expanded on the earlier work of Pezzoli *et al.* (2000) and Racette *et al.* (2001). Although Pezzoli *et al.* (2000) and Racette *et al.* (2001) provided evidence that age at onset of PD is influenced by occupational exposure to chemicals both studies failed to specifically control for known heritable genetic risk factors that may influence age at onset of PD by excluding subjects with a family history of the disease.

The influence of genetic factors on age at onset of PD were minimized in this study access the influence of metals and pesticides on age at onset of PD by excluding subjects with a positive family history of the disease among their first-degree relatives. Because genetic factors can influence age at onset of PD as well as lifetime risk for the disease, family history of PD must be considered when interpreting data related to age at onset of the disease. The greater similarity for age at onset than for year at onset among siblings with PD together with an increased risk for the disease among subject's biological relatives compared with subject's spouses stresses the importance of controlling for heritable genetic components (Maher *et al.*, 2002). Although we could not control for all genetic variables in this retrospective study, the results presented here nevertheless suggest that age at onset of PD is influenced by occupational exposure history in the absence of recognized heritable genetic risk factors for the disease.

The genetic factors that were able to control for in this study were those associated with a positive family history of the disease including: the ubiquitin C-terminal hydrolase gene located on chromosome 4p14/PARK5; the α-synuclein gene on chromosome 4q21-23/PARK1; and, the parkin gene located on chromosome 6q25-27/PARK2. α-Synuclein aggregation may be involved in Lewy body formation and in the pathogenesis of autosomal dominant forms of familial PD (Polymeropoulos *et al.*, 1997). The ubiquitin C-terminal hydrolase gene located on chromosome 4p14/PARK5 has been associated with autosomal dominant PD (Leroy *et al.*, 1998).

Unfortunately, even by excluding subjects with a positive family history of PD we could not fully control for the influence the parkin gene (Kitada *et al.*, 1998; Periquet *et al.*, 2003). Parkin mutations have been associated with about 15% of early-onset cases (≤45 years-old) without a family history of PD; this proportion decreased significantly with increasing age at onset (Periquet *et al.*, 2003). Because it is conceivable that some of the subjects with no family history in our sample population might still have the parkin gene mutation, future studies to control for this recessive genetic variable are recommended.

This study relied on retrospective data provided by the subjects about the medical history of their first-degree relatives. The lack of actual medical record reviews to confirm the diagnoses should be recognized as a potential source of error. However, because this same method for subject selection has been used extensively in the GenePD study we therefore deemed it reliable for the purposes of this study as well (Maher *et al.*, 2002; DeStefano *et al.*, 2002).

#### Bias

Because the subjects evaluated in this study were selected first on the basis of familial history of PD selection bias was minimized and not based on exposure history per se selection bias was minimized. Occupational exposure history data was ascertained by a blinded interviewer who was not involved in the subject selection process nor in data analysis thereby further minimizing potential for selection bias. Stratification of subjects was performed by the PI but was based on exposure history alone with no regard for age at onset. All data on age at onset was ascertained after subjects were stratified by exposure history.

## Conclusions

The results of this study suggest that environmental factors such as occupational exposure to metals and pesticides can influence age at onset of sporadic PD. This study is the first to demonstrate an association between occupational exposures to chemicals while attempting to specifically control for the influences of heritable genetic factors by excluding subjects with a positive family history of PD among first-degree relatives.

The results of this study also suggest that exposure duration is negatively correlated with age at onset of PD. However, because 85% of the variance in age onset of PD could be accounted for by other factors such as intensity of exposure, additional studies are needed to better define this relationship.

The observation that smoking is associated with an older age at onset among subjects with sporadic PD regardless of exposure history is intriguing. Additional research is needed to determine how smoking history differentially influences age at onset among subjects with sporadic and familial forms of PD.

The data from this study along with the previous findings of Racette *et al.* (2001) and Pezzoli *et al.* (2000) suggest that a common mechanism of action may underlie the effects of occupational chemical exposure on age at onset of PD. Because oxidative stress has been associated with PD and many of the chemicals these subjects were potentially exposed to may increase oxidative stress it is conceivable that increased oxidative stress is the common mechanism which may have hastened the progression of PD in these subjects. Future studies could be developed to test this hypothesis by ascertaining how genetic polymorphisms that influence the activity of enzymes such as glutathione-*S*-transferase which are involved in the metabolism and detoxification of neurotoxicants interact with exposure history to influence age at onset of sporadic PD (Offen *et al.*, 1996; Seaton *et al.*, 1997; Shimoda-Matsubayashi *et al.*, 1997; Menegon *et al.*, 1998; Feldman & Ratner, 1999; Dawson & Dawson, 2003). Studies looking at age at onset of PD may help to provide information about the etiology as well as the progression of this disabling disease and may contribute to the development of novel preventative and therapeutic strategies.
